# Dynamic pressure analysis of novel interpositional knee spacer implants in 3D-printed human knee models

**DOI:** 10.1038/s41598-022-20463-6

**Published:** 2022-10-07

**Authors:** Korbinian Glatzeder, Igor Komnik, Felix Ambellan, Stefan Zachow, Wolfgang Potthast

**Affiliations:** 1 Institute of Biomechanics and Orthopaedics, German Sport University Cologne, Am Sportpark Müngersdorf 6, 50933 Cologne, Germany; 2 Zuse Institute Berlin (ZIB), Takustraße 7, 14195 Berlin, Germany; 3 Freie Universität Berlin, Kaiserswerther Str. 16-18, Berlin, Germany

**Keywords:** Bone, Cartilage, Biomedical engineering, Mechanical engineering

## Abstract

Alternative treatment methods for knee osteoarthritis (OA) are in demand, to delay the young (< 50 Years) patient’s need for osteotomy or knee replacement. Novel interpositional knee spacers shape based on statistical shape model (SSM) approach and made of polyurethane (PU) were developed to present a minimally invasive method to treat medial OA in the knee. The implant should be supposed to reduce peak strains and pain, restore the stability of the knee, correct the malalignment of a varus knee and improve joint function and gait. Firstly, the spacers were tested in artificial knee models. It is assumed that by application of a spacer, a significant reduction in stress values and a significant increase in the contact area in the medial compartment of the knee will be registered. Biomechanical analysis of the effect of novel interpositional knee spacer implants on pressure distribution in 3D-printed knee model replicas: the primary purpose was the medial joint contact stress-related biomechanics. A secondary purpose was a better understanding of medial/lateral redistribution of joint loading. Six 3D printed knee models were reproduced from cadaveric leg computed tomography. Each of four spacer implants was tested in each knee geometry under realistic arthrokinematic dynamic loading conditions, to examine the pressure distribution in the knee joint. All spacers showed reduced mean stress values by 84–88% and peak stress values by 524–704% in the medial knee joint compartment compared to the non-spacer test condition. The contact area was enlarged by 462–627% as a result of the inserted spacers. Concerning the appreciable contact stress reduction and enlargement of the contact area in the medial knee joint compartment, the premises are in place for testing the implants directly on human knee cadavers to gain further insights into a possible tool for treating medial knee osteoarthritis.

## Introduction

Osteoarthritis (OA) is a common condition most frequently affecting the knee joint, with few operative treatment options^1^. Major health implications arise because of two mutually dependent factors: (1) the increase of OA with age and (2) a decreasing physiological function^2^. Degeneration of the articular cartilage can significantly change the load distribution in the knee^3^ leading to increased peak loads, higher joint contact stresses, and risk of joint degeneration^4,5^. Arthroscopic procedures like arthroscopic debridement and arthroscopic partial meniscectomy^6^, osteotomy^7,8^, and unicompartmental knee replacement (UKR) suitable surgical choices for young patients (under 50 years) with severe knee-OA^9^ if they suffer from unicompartmental OA^10^. Hemiarthroplasty implanted in patients initially by MacIntosh and McKeever was intended to prevent the articular cartilage of the medial compartment from further degeneration^11^. Based on this idea, the derivative, a so called spacer is placed between the femoral condyle and the tibial plateau on the medial side of the knee and is supposed to restore the stability of the knee, to correct the malalignment of a varus knee, to reduce pain and peak strains, and to improve joint function as well as gait^12^. The spacer implant is intended to enlarge the contact surface of the femoral condyle in the medial compartment and prevent the joint partners from more mechanical damage due to friction and further mechanical stresses. A spacer has the potential advantage of preserving the natural bone stock until a more invasive treatment method is needed, without compromising the patient’s future knee replacement and offering relief of OA-associated knee pain and improved joint function.

The lack of acceptance of using previously developed metallic and synthetic spacers as treatment options is primarily due to persisting pain and lack of long-term outcomes^13^. Research is limited in developing anatomically -based spacer shape design, including proper materials and in vitro biomechanical testing under physiological loading and kinematic aspects^11,14,15^. To address the aforementioned requirements, a collaboration of an interdisciplinary consortium consisting of scientists with expertise in material sciences, engineering, medical image, and geometry analysis, implant technology, and clinics, developed novel, elastic, interpositional knee spacer implants.

The purpose of this study was to provide a biomechanical analysis, including the verification of the general functional principal of the newly developed spacers, under dynamic loading conditions. Since high levels of contact stress are associated with accelerated OA progression^16^, the main goal of the spacers was to mitigate peak contact stresses by distributing the acting force to a larger area. It was assumed that an anatomically-adapted spacer shape better conforms to the knee morphology and would allow for self-centering during movements.

The influence of different knee spacers on knee joint loading parameters was investigated using a knee joint loading simulator in combination with a set of six 3D printed knee models reproduced from cadaveric leg CTs. It was presumed that the spacers distribute the acting forces to a larger contact area, resulting in reduced pressure values. Furthermore, the extent of pressure reduction was expected to be dependent on the interaction of the respective spacer geometry and the geometry of the individually reproduced knee model.

## Methods

Six 3D-printed human knee models consisting of distal femur, proximal tibia, and patella were used in this investigation The artificial 3D knee models correspond to six fresh-frozen right-leg specimens (mean age: 82.8 years old; sex: 3 male, 3 female; informed consent was obtained from all subjects) with mild to severe medial osteoarthritis. The body donors were used with approval from the ethical review committee of the German Sport University Cologne, all methods were performed in accordance with the relevant guidelines and regulations. The legs were scanned using a CT scanner (SOMATOM Emotion eco, Siemens Healthcare GmbH, DE, slice thickness: 0.6 mm). All six knees were geometrically reconstructed by manual bone segmentation and surface reconstruction using Amira ZIB Edition (Zuse Institute Berlin, DE). The attachment-site centers of the anterior cruciate ligament (ACL) and posterior cruciate ligament (PCL) were identified and marked on the reconstructed bone geometries. CAD models, i.e. Non-Uniform Rational B-Spline Surfaces (NURBS), were generated from triangulations of the segmented bone parts (STL) using an inverse engineering approach in Power Surfacing for SolidWorks® (SolidWorks® 2016, Dassault Systèmes, FR). This allowed the investigators to modify the CAD knee models to be fixed in custom-made holders. Polylactic Acid (PLA) served as printing material for an extrusion-based 3D printer (X400 Pro V1, German RepRap GmbH, DE). PLA, along with ABS, is one of the most commonly used materials in FDM printers. These polylactides are synthetic polymers that belong to the group of polyesters and are made up of many chemically bonded lactic acid molecules. The material has high chemical resistance and high surface hardness.

**Figure 1:**
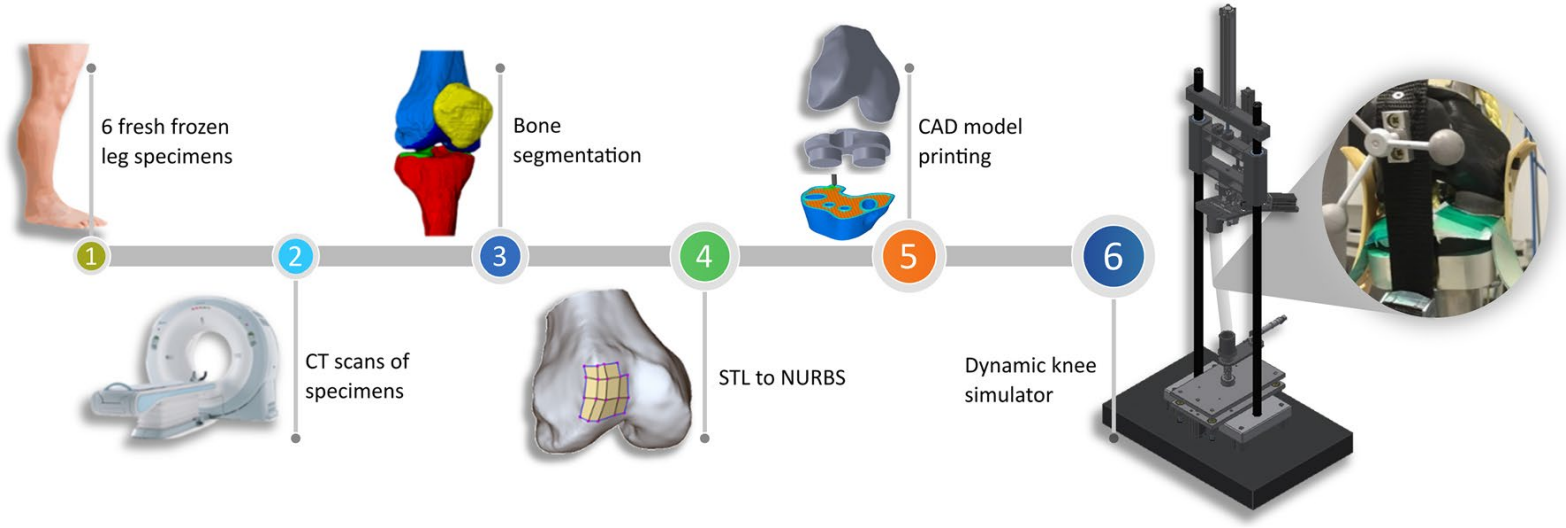
Depiction of the methodological workflow. After scanning every leg specimen (1,2), the different knees were geometrically segmented (3) to further process the data (4) towards printing the articulating knee parts (5) and implement them into the dynamic knee loading simulator (6) (*CT* computed tomography, *STL* standard triangle language, *NURBS* non-uniform rational B-spline, *CAD* computer aided design).

The 3D-printed femora, tibiae, and patellae were mounted in a dynamic knee simulator, mimicking knee flexion–extension motion with a constant angular velocity of 17°/s and mechanically induced muscle forces. All six degrees of freedom of the knee were constituted by inserting the femur metal shaft to a ball joint which was fixed at a rotatable plate on the traverse of the simulator. The flexion–extension movement of the artificial knee was initiated by a traverse which could move down and up in vertical direction. The tibial metal shaft was mounted on a cylindrical joint to generate the mobility of the ankle joint (Fig. 1). The anterior and the posterior cruciate ligaments (ACL, PCL), the medial collateral ligament (MCL), the lateral collateral ligament (LCL), and the triceps surae muscle (MTS) were artificially recreated and implemented in the mechanical knee model. Since the meniscus of a human knee must be resected when inserting a spacer, the model does not present a meniscus. Muscle forces were induced by pneumatic actuators in order to simulate internal joint forces: the quadriceps muscle pulled with a constant force of 700 N during the flexion–extension knee motion, while the medial and lateral hamstrings exerted a constant force of 250 N, simultaneously^20^. A Teflon rope was attached to the insertion of the patella tendon to simulate the quadriceps muscle, and two to the ischium for the anatomical origins of the hamstring muscles.

Joint contact stresses (JCS) and joint contact areas (JCA) were measured using thin and flexible Tekscan pressure sensor sheets (K-Scan™, PMS 4000, 900psi, Tekscan Inc., US). The width of the sensing matrix on every compartment is 27.9 mm and the height is 33.0 mm. Each sensor was calibrated and equilibrated using a ZWICK system (Zwick 020, Zwick GmbH & Co. KG, DE). The Sensor calibration was conducted by applying the Two Point Power Law calibration with regard to the manufacturer’s recommendations prior to testing^21^. The manufacturer’s software TestXpert V10.0 was used to create the loading protocols.

**Figure 2:**
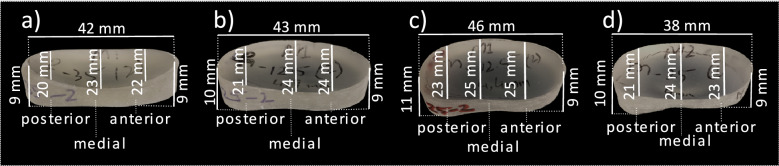
Spacer dimensions. Depiction of the four different spacers A–D and their dimensions.

Four different spacers (Fig. 2) were developed and manufactured (be innovative GmbH, Cologne, Germany) in a polyurethane (PU) pressing process using their own aliphatic polyurethane (shore-A hardness 70–73). To determine what spacer designs best fit the anatomical range of the study population, a form-finding process was deduced using SSM of anatomical knees derived from the OAI database^22^. A subset of 507 datasets, representing all grades of OA according to the Kellgren & Lawrence^23^ classification was selected and segmented using an SSM-based approach, trained on the public SKI10 dataset^24^. All segmentations were carefully revised and manually corrected in cases where necessary. The resulting segmentation masks (femur, tibia as well as the respective cartilage) were made publicly available as an OAI-ZIB dataset^25^. Further details on demographic aspects and segmentation procedures can be found in Seim et al.^26^ and Ambellan et al., respectively^25^. The SSMs for all bones were generated via a consistent surface decomposition and a common parametrization resulting in a set of corresponding vertices for each bone segment^27^. These sets of vertices for all knees were transformed via principal component analysis into a point distribution model that is represented by the mean geometry of the respective anatomical structure together with statistical modes of geometric variation^19^.

**Figure 3:**
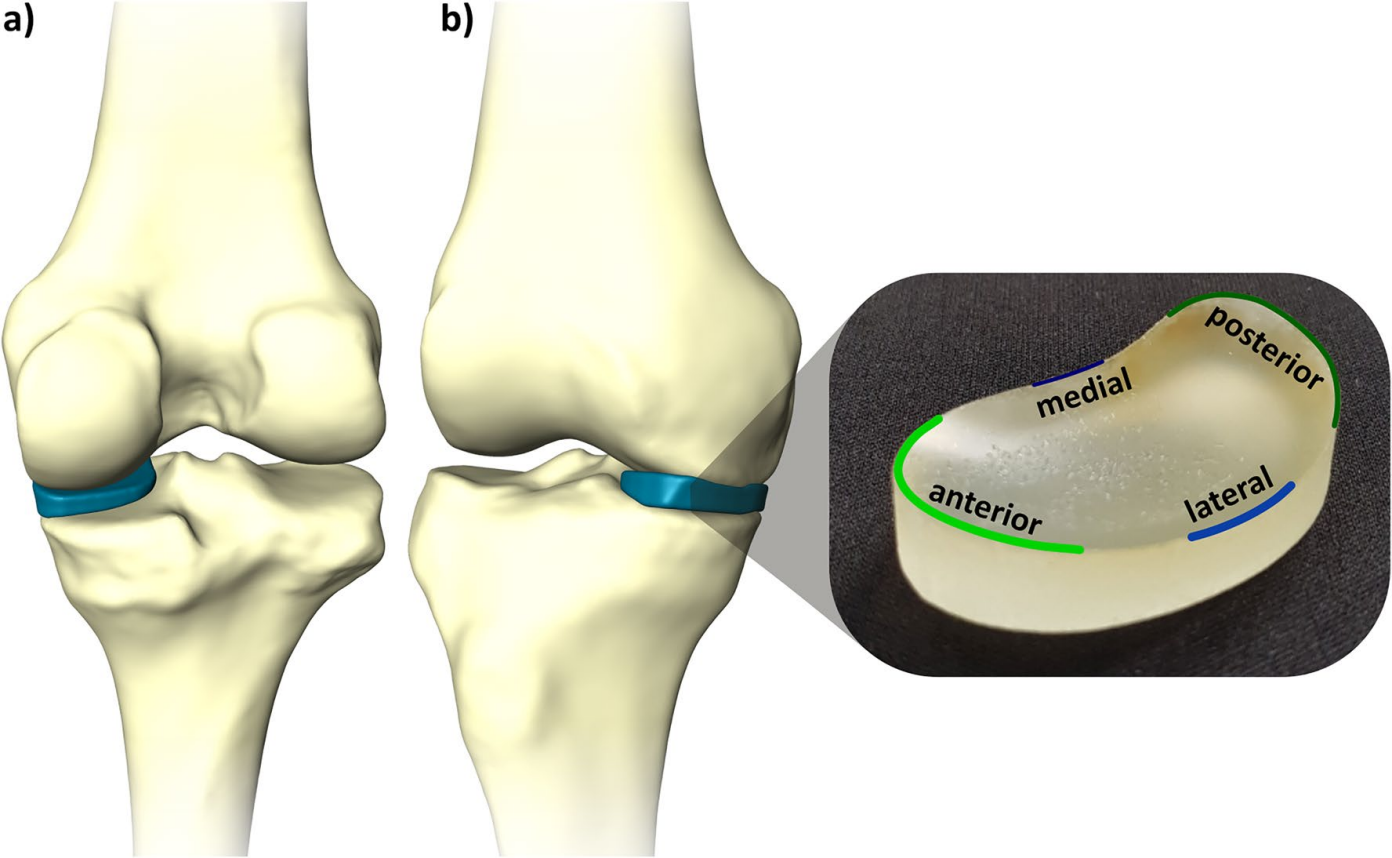
Knee spacer implant. An example of a knee spacer template filling the medial joint gap depicted in posterior and anterior view.

The determined SSM mean shape knee model was used to fit a corresponding spacer shape in the medial knee joint gap by the planning tool. Since variations in the spacer can have different effects on the distribution of loads, four variations were created based on the mean shape. The four spacers are reniformed to prevent tilting at the Eminentia Interkondylaris (Fig. 3). Specifically, they can be divided into two groups to differentiate: spacer C and D are designed for larger knee geometries and are larger in length, width, and thickness than the other two spacers (spacer A and B). Regardless, all of the four spacers vary in that the planes of the bottom and top are inclined differently from one another. All spacers are concave on the top and bottom. The rear and front parts of the spacer surfaces show a different elevation and a different gradient angle of the elevation. These differences in the spacer geometry are intended to show in the application whether there are differences in the effect of the pressure distribution.

Prior to testing the four spacers in every artificial knee geometry, the 3D printed knee models were mounted into the joint loading simulator and the muscle tracts were attached to the corresponding anatomical landmarks. Tekscan sensors were inserted anteriorly to posteriorly on the medial and lateral side of the tibial plateau, between the articulating surfaces of the femur and tibia. An adhesive tape was stretched on the bottom of the sensors and attached to the 3D printed tibial plateaus to ensure the fixation of the sensors throughout the testing procedures.

To investigate the effects of the spacers in the different knee geometries in simulated dynamic flexion–extension movements, each of the four spacer templates was inserted in the medial condyle of each 3D printed knee model (Fig. 3). Furthermore, an additional condition was performed with no spacer inserted. Data was collected at 50 Hz (I-Scan System, Tekscan Inc., US). The average of 27 flexion–extension cycles was used for the analysis within a movement range of 5–90°. Pressure data was time-normalized to the flexion–extension phase. Data processing was conducted with custom-built Matlab (2019a) routines (The MathWorks, Natick, USA). Force and area were calculated by reference to the tekscan pressure maps (F = kPa*area; area = sum of the loaded Tekscan cells. Force peak: Peak force/Frame. Mean stress: (Sum stress/loaded cells)/Frame).

Descriptive statistics are presented in this study for all considered variables due to the small sample size, including the average values for maximum peak pressure (MPP) and mean pressure (MP), contact force, and area of loaded sensors, as well as the corresponding standard deviations. Furthermore, 95% confidence intervals (CI) were calculated for the no-spacer condition based on the one-dimensional time series analysis proposed by Pataky et al.^28^ using the open-source spm1d package (Version M.0.4.5, www.spm1d.org) in Matlab (2019a).

### Ethics statement

Ethics were approved by the ethical review committee of the German Sports University Cologne.

## Results

All spacer types showed reduced mean stress values by 84–88% in the medial knee joint compartment compared to the non-spacer test condition (Table 1).

**Table 1:** Discrete stress, area and force parameters, mean and standard deviation are presented.

Knee compartment	Mean stress (MPa)	sd	Spacer-type	Difference (%) no-spacer vs all spacer types
Medial	1.1	0.6	Spacer-A	− 84
	0.8	0.3	Spacer-B	− 88
	0.9	0.2	Spacer-C	− 86
	0.9	0.3	Spacer-D	− 86
	6.8	6.3	No-spacer	
Lateral	3.0	1.5	Spacer-A	− 60
	3.5	0.4	Spacer-B	− 54
	3.4	0.3	Spacer-C	− 55
	3.5	0.5	Spacer-D	− 53
	7.5	3.1	No-spacer	

Knee compartment	Peak stress (MPa)	sd	Spacer-type	Difference (%) no-spacer vs all spacer types
Medial	4.0	2.8	Spacer-A	− 617
	4.1	0.8	Spacer-B	− 602
	4.6	1.0	Spacer-C	− 524
	3.6	0.8	Spacer-D	− 704
	28.9	20.7	No-spacer	
Lateral	7.1	3.6	Spacer-A	− 83
	8.7	1.0	Spacer-B	− 79
	8.8	0.9	Spacer-C	− 79
	8.8	0.9	Spacer-D	− 79
	42.3	21.6	No-spacer	

Knee compartment	Area (mm^2^)	sd	Spacer-type	Difference (%) no-spacer vs all spacer types
Medial	452	233	Spacer-A	462
	584	67	Spacer-B	627
	532	78	Spacer-C	563
	584	57	Spacer-D	627
	80	27	No-spacer	
Lateral	111	70	Spacer-A	5
	145	29	Spacer-B	37
	138	32	Spacer-C	30
	127	24	Spacer-D	19
	106	39	No-spacer	

Knee compartment	Force (N)	sd	Spacer-type	Difference (%) no-spacer vs all spacer types
Medial	472.2	262.1	Spacer-A	68
	412.3	184.7	Spacer-B	47
	413.3	141.6	Spacer-C	47
	398.8	132.5	Spacer-D	42
	280.8	110.4	No-spacer	
Lateral	312.3	182.8	Spacer-A	− 23
	371.3	52.0	Spacer-B	− 9
	347.4	82.4	Spacer-C	− 15
	352.5	75.6	Spacer-D	− 13
	406.5	168.3	No-spacer	

**Figure 4:**
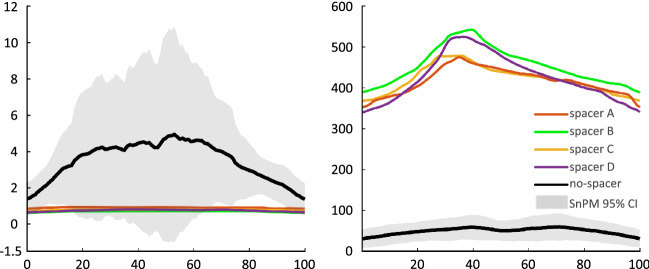
Mean stress and loaded area. Time series representing a) mean stress and b) loaded area (± 95% confidence interval of the no-spacer condition calculated using statistical non-parametric mapping (SnPM)).

The course of stress progression of all spacer types clarifies that the mean values were drastically diminished during the entire flexion–extension cycle compared to the condition with no medially inserted spacer (Fig. 4a). Peak stress was reduced in all spacer conditions by 524–704% compared to the non-spacer conditions in the medial joint compartment and by 79–83% in the lateral compartment (Table 1).

**Figure 5:**
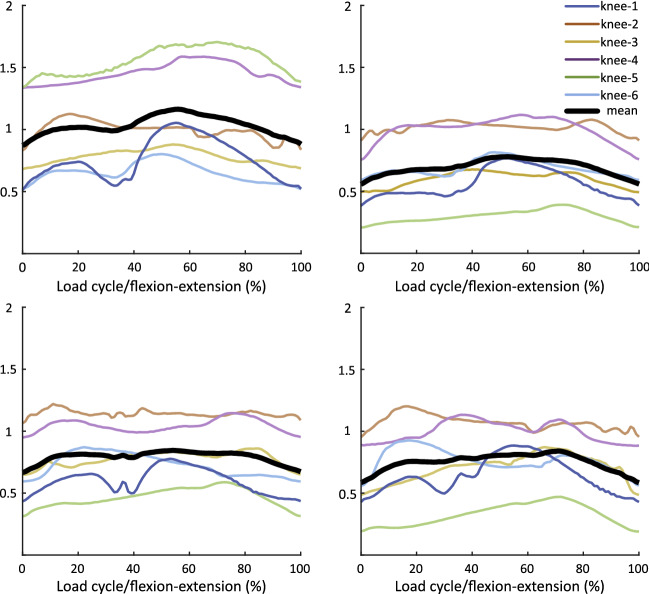
Mean stress progression. Time series representing mean stress progression of the individual tested knee models (1–6) with the corresponding spacer type.

The time series of the corresponding spacer type exceeded the continuous 95% confidence interval lower bounds extracted from SnPM during 3–18% (CI 0.6 MPa) and 66–99% (CI 0.7–0.6 MPa) of the knee-flexion cycle. Generally, a wide 95% confidence interval could be observed in the no-spacer condition represented by the grey shaded area in Fig. 4a, ranging from − 1 to 10.89 MPa. In this regard, knee model No. 1 (knee-1) revealed appreciable increased mean stress time series in conjunction with decreased loaded mean area, due to relatively prominent geometry of the femoral condyles and tibial plateaus compared to the other examined knee models. A better form fit between the articulating joint surfaces was achieved with the use of the spacer. This should also lead to diminished translation in the flexion–extension movement. A quantitative evaluation of Fig. 5 suggests that each spacer type can substantially lower the stress magnitudes in knee-1 to the mean stress level, concerning all individual knee models.

**Figure 6:**
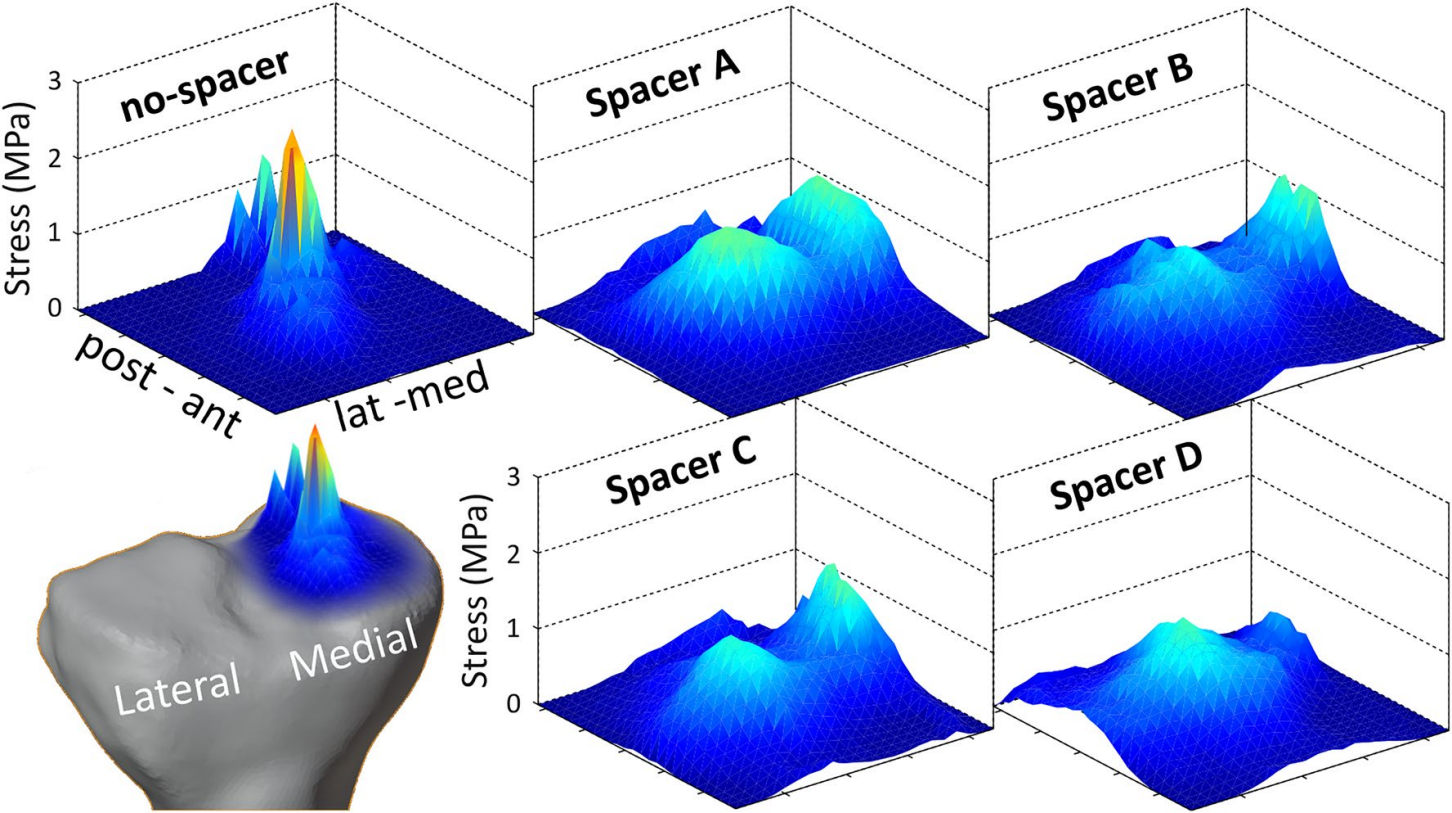
Heat map. Mean stress heat map of the medial knee condyle for no-spacer and four tested spacer conditions.

The contact area was enlarged with the tested spacers by 462–627% compared to the non-spacer condition in the medial knee condyle with slightly increased force values by 42–68% (Table 1, Fig. 4b). The stress heat map (Fig. 6) additionally illustrates the redistributed force to a larger area using a medially inserted spacer type. The u-shaped mean stress distribution in the mediolateral direction during the knee flexion–extension cycles (Fig. 6: Spacer A, B, C) indicates the presence of the intended interpositional property.

## Discussion

The primary outcome in the current study demonstrates an appreciable reduction of mean stresses by 84–88% as well as diminished peak stress magnitudes by up to 704% in the medial compartment of the knee joint with the inserted knee spacers. Of note, the stress reduction was observed to the same amount in every tested spacer type. Based on the outcomes of this study, two major beneficial clinical implications arise from utilizing spacers in patients suffering from knee OA in the medial joint compartment. Firstly, high peak stresses which are partly responsible for the worsening of OA can be reduced with the help of a spacer^16,29,30^. The results clearly confirm the presumed functional principle of a knee spacer, namely to enlarge the articulating load area in the medial knee compartment, thereby reducing mean and peak stresses. Secondly, the clinical relevance shows that reducing mean and peak contact stresses may result in a temporary improvement of patients’ OA symptoms^31^.

From a mechanical point of view, a high level of contact stress-inducing critical strain magnitudes is one of the driving factors for OA, which is not only associated with the initiation of a symptomatic knee OA, but also with the progression of the disease^16,29,30,32^. Relatively high peak stresses and stress rates that occur close to the already damaged cartilage matrix can increase the risk of OA progression due to geometrical incongruence of focal articular defects^16^. Reducing the accumulated stress and peak stress with the help of a spacer can slow down further cartilage damage and postpone more severe surgical treatment options by several years. Moreover, a previous study by Hurwitz et al.^33^ revealed that decreases in pain are linked to increased knee joint loading in patients with medial knee OA. The inverse correlation was presumed to be a pain protective mechanism in response to increased joint loads induced by moments acting in frontal and/or sagittal planes. Thus, a further connected intention of the introduced knee spacers or their future variations is to relieve OA associated knee pain. As a result, joint function can be improved in the subgroup of patients who tend to unload their affected knee joint due to pain. Being aware that the connection between pain, quadriceps weakness, and OA progression is discussed controversially^34^, there is evidence that clarifies the positive relation between contact stress and symptomatic knee OA^31^.

Treatment of particularly younger and active knee OA patients (< 65 years) can be challenging with different types of knee arthroplasties in terms of patients’ preoperative degree of expectations, patient selection, joint mechanics, and long-term survival^35–37^. Total knee replacement (TKR) serves as a standard procedure after failed UKR. However, changing to TKR after a surgical correction of the mechanical axis alignment by UKR or HTO results in more difficult operating conditions, and less lifetime of the replacement. Consequently, higher revision rates and poorer outcome scores may result^38–40^. Hemiarthroplasty is a further surgical treatment for severe OA reported and implanted in OA patients initially by MacIntosh and McKeever^11^. Building on the aforementioned drawbacks of the established surgical treatments, it gives rise to develop alternative metallic implants. Few publications to date examine knee spacers and their basic functional principles. The UniSpacer™ (Centerpulse Inc, US), as a representative of a metallic spacer, showed a high rate of failure between 16 and 44% mainly due to persisting pain^11,14,15,41^, and was not recommended by several authors. The early results using the iForma™ (iForma™, ConforMIS Inc., USA) knee implant were promising but did not illustrate long-term outcomes^13^. The NUsurface® Meniscus Implant (ACTIVE IMPLANTS LLC, US) represents the new era of knee spacers, consisting of a compliant polycarbonate-urethane matrix reinforced with high modulus ultrahigh molecular weight polyethylene (UHMWPE) fibers. In contrast to the metallic knee spacers, this implant can adapt to the joint shape. Nevertheless, it was not tested under physiological loading^42^ and the results of the implants did not live up to expectations. Even though significant pain relief and improvements in clinical as well as functional outcomes could be observed, the overall short-term results were rather poor. Most notable were failure rates of up to 44%, primarily due to persisting pain and in some cases due to dislocation of the implants^14,43,44^. The authors associated implant size matching and incongruence between the femur condyle and implant articulating surface with the high revision rates. To counteract the implant design-related issues, Brooks et al.^13^ investigated a patient-specific metallic implant revealing also high failure rates of 19% within the first year. Pain was listed as the main source for revision surgeries, whereas no implant dislocations were found which is presumably connected with the patient-specific manufacturing of the implants^13^. Persisting pain might be the consequence of the inability of the hard and rigid cobalt-chrome to diminish focal stress in the OA-affected region^45^. Since spacers made of metal did not meet expectations^14,43,44^, interpositional spacers made of PU were preferred^45^. Compared to UHMWPE, PU has superior wear resistance, i.e. better abrasion properties^46^, and similar to biological meniscus a non-linear viscoelastic behavior during cyclic loading^42^. As well, it can be assumed that softer PU material with a shore hardness below 85 is rather capable to enlarge the articulating surface than metallic implants. This would imply a greater reduction in joint peak load compared to cobalt chrome implants. The resulting pain relief is also beneficial, especially about the fact that pain is the main cause of implant failure^33^. Additionally, the deformable property of a PU spacer allows its shape to be adapted to the morphology of the rounded femoral condyle and to improve the self-centering mechanism, hence reducing the risk of spacer dislocation. However, the use of state of the art medical PU as material for spacers is also associated with several downsides. Of note, the fluid uptake that softens the PU and therewith influences the long-term biostability^47^. On the other hand, the absorption of joint fluids can improve lubrication and ultimately diminish PU wear rates^48^. About the enlargement of the articulating load area, the results of the current study indicate that the lubricating layer will be enhanced due to reduced contact stresses^48^, which consequently will result in improved longevity of the knee spacers.

Following the aforementioned problems associated with polymer materials such as PU, it is observed that polymer materials breakdown as a result of mechanically loaded arthroplasties can induce inflammatory reactions of the adjoining joint tissue^49^. This aspect is particularly relevant considering the use of an interpositional knee spacer in OA patients since wear debris will not only occur due to the abrasion on the enlarged articulating area but also due to backside wear at the inferior surface^50,51^. Taking into account the adverse consequences associated with the accumulation of wear particles, there is evidence that the inflammatory response is lower in comparison to UHMWPE^15,52^. PU has also been tested positive for its durability in stress studies^53,54^.

The appropriate choice of the spacer material is only one aspect to provide an effective orthopedic implant. Spacer geometry of the articulating surface is the other, which is crucial to mitigating knee OA symptoms and reducing the risk of spacer dislocation. In this study, the form-finding process was derived from using SSM of anatomical knees from the OAI database^22^. With this approach of determining the geometry of the spacers, the newly developed PU spacers differ significantly from the alternatives available on the market.

Even though all spacer types reduced the mean and peak stresses to a similar extent (Figs. 4a, 5), it is apparent from Fig. 5 that certain spacer types performed more effectively than others when considering the stress curve in each individual knee model. It was observed that wider spacers (like spacer C) showed better adaptation to the femoral condyle. However, a smaller knee geometry (like spacer A) must be taken into account as wider implants are at disadvantage due to the limitation of space, especially caused by the elevation of the eminentia intercondylaris. Figure 6 illustrates that the medial and lateral edges of smaller spacers are more heavily loaded in larger knee geometries, which can promote dislocation of the spacer, demonstrating that a single universal spacer will not work in all knee morphologies. The suggested design approach will address the persisting pain and spacer dislocation issue, minimizing the risk of protruding the gap in anterior–posterior, as well as medial–lateral direction, on the one hand. On the other hand, creating a selection of different spacers based on population-based customization makes it feasible to create an affordable product. Even though highly precise and automatic segmentation methods for knee MRIs are available^25^, a subject-specific production of the presented spacers still does not render possible because of certification processes. Due to the manufacturing process of the PU, its material properties and storability do not allow a short-term creation of the implants.

Thus, it can be concluded that prefabricated spacers enable the surgeon to find the right selection for each patient without the need for expensive custom-made spacer implants. Considering automatic geometry reconstruction of the involved bones, as well as their positioning relative to each other, comprise a reasonable benefit of an automated spacer pre-selection (from a customized set of prefabricated implants) that optimally adapts to the articulating surfaces. Initial concepts have already been presented^55^ and could pave the way to a more precise patient-specific surgery planning, aiming for further maximizing the positive outcome of surgical treatments.

Several limitations in connection with the utilized methodological approach and resulting outcomes of the current study need further discussion. First, the knee spacers were tested in a dynamic knee simulator pulling with a resultant force of 950 N. The resultant forces presented by Kutzner et al.^56^ exceeded magnitudes of 250% body weight during level walking and 340% bodyweight during stair descent in patients with instrumented total knee arthroplasties. Considering the bodyweight of the OA population^57^, the induced forces and consequently the contact stresses are underestimated. Nevertheless, the presented functional principle of the knee spacers is applicable in knee joints exposed to higher forces, whereas such higher forces also imply a higher risk of spacer dislocation, particularly during activities of daily living at relatively high knee flexion angles. Furthermore, the 3D-printed knee models extruded of PLA do not meet the mechanical properties, e.g. strength and stiffness, of biological cartilage or bone respectively^58,59^, implying that stress magnitudes are likely overestimated. This leads to the presumption that the considerable difference in stress magnitudes between the spacer and no-spacer conditions would be lower in biologic knee joints. The results of the current study should be interpreted with caution due to the descriptive statistical analysis which does not imply hypothesis testing using inferential statistics.

The results show that the articulating joint partners move further away from each other, instead of developing a capping of the knee joint to the lateral side. A change in the pressure distribution was obtained, however, the distribution of force lateral and medial was the same. Measurements with spacer showed a reduced decrease in force (10–20%), whereas the decrease was higher on the medial side of the knee joint (25–40%).

Although hemiarthroplasty was introduced decades ago, biomechanical investigations analyzing the fundamental functional principle behind knee spacers as an OA treatment under realistic geometric conditions had not been carried out to this date. To our knowledge, this is the first study to investigate the functional principle of interpositional knee spacers made of PU under loading with realistic arthro-kinematics including dynamic compression tests in a custom-built knee joint loading simulator. From a biomechanical testing perspective, it is necessary to consider cadaveric specimens, more realistic muscle forces, and kinematic analysis in future studies. In closing, the results of the present study conclude that the spacer implants reduced mean stress values in the artificial knee joint up to 84–87% and extend the contact area by 462–704%.
