# Author Correction: Dynamic pressure analysis of novel interpositional knee spacer implants in 3D-printed human knee models

**DOI:** 10.1038/s41598-022-26540-0

**Published:** 2022-12-19

**Authors:** Korbinian Glatzeder, Igor Komnik, Felix Ambellan, Stefan Zachow, Wolfgang Potthast

**Affiliations:** 1 Institute of Biomechanics and Orthopaedics, German Sport University Cologne, Am Sportpark Müngersdorf 6, 50933 Cologne, Germany; 2 Zuse Institute Berlin (ZIB), Takustraße 7, 14195 Berlin, Germany; 3 Freie Universität Berlin, Kaiserswerther Str. 16‑18, Berlin, Germany

Correction to: *Scientific Reports* 10.1038/s41598-022-20463-6, published online 07 October 2022

The original version of this Article contained an error in the name of author Igor Komnik, which was incorrectly given as Komnik Igor.

The original Article has been corrected.

